# Conserved and unique features of the homeologous maize Aux/IAA proteins ROOTLESS WITH UNDETECTABLE MERISTEM 1 and RUM1-like 1

**DOI:** 10.1093/jxb/erv519

**Published:** 2015-12-15

**Authors:** Yanxiang Zhang, Caroline Marcon, Huanhuan Tai, Inga von Behrens, Yvonne Ludwig, Stefan Hey, Kenneth W. Berendzen, Frank Hochholdinger

**Affiliations:** ^1^Center for Molecular Cell and Systems Biology, College of Life Science, Fujian Agriculture & Forestry University, 350002 Fuzhou, China; ^2^Crop Functional Genomics, Institute of Crop Science and Resource Conservation, University of Bonn, 53113 Bonn, Germany; ^3^ZMBP, Center for Plant Molecular Biology, General Genetics, University of Tuebingen, 72076 Tuebingen, Germany; ^4^ZMBP, Center for Plant Molecular Biology, Central Facilities, University of Tuebingen, 72076 Tuebingen, Germany

**Keywords:** Aux/IAA, maize, protein interaction, root, RAP1, RUM1, RUL1.

## Abstract

The maize (*Zea mays* L.) Aux/IAA protein RUM1 (ROOTLESS WITH UNDETECTABLE MERISTEM 1) is a key regulator of lateral and seminal root formation. An ancient maize genome duplication resulted in the emergence of its homeolog *rum1-like1* (*rul1*), which displays 92% amino acid sequence identity with RUM1. Both, RUL1 and RUM1 exhibit the canonical four domain structure of Aux/IAA proteins. Moreover, both are localized to the nucleus, are instable and have similar short half-lives of ~23min. Moreover, RUL1 and RUM1 can be stabilized by specific mutations in the five amino acid degron sequence of domain II. In addition, proteins encoded by both genes interact *in vivo* with auxin response factors (ARFs) such as ZmARF25 and ZmARF34 in protoplasts. Although it was demonstrated that RUL1 and RUM1 can homo and heterodimerize *in vivo*, *rul1* expression is independent of *rum1*. Moreover, on average *rul1* expression is ~84-fold higher than *rum1* in the 12 tested tissues and developmental stages, although the relative expression levels in different root tissues are very similar. While RUM1 and RUL1 display conserved biochemical properties, yeast-two-hybrid in combination with BiFC experiments identified a RUM1-associated protein 1 (RAP1) that specifically interacts with RUM1 but not with RUL1. This suggests that RUM1 and RUL1 are at least in part interwoven into different molecular networks.

## Highlight

The maize Aux/IAA protein RUM1 and its homeolog RUL1 display conserved biochemical properties. The specific interaction of RAP1 with RUM1 but not RUL1 suggests differences in their molecular interactions.

## Introduction

Maize (*Zea mays* L.) plays an important role as fodder, human food and a source of bioethanol. The maize root system is formed by embryonic primary and seminal roots and post-embryonic shoot-borne crown and brace roots, which are instrumental for water and nutrient uptake and for anchorage of plants in soil (Hochholdinger and Tuberosa, 2009). All these root-types form post-embryonic lateral roots, which are initiated from pericycle and endodermis cells and make up the major backbone of the plant (Hochholdinger *et al.*, 2004).

The phytohormone auxin is a key regulator of almost all developmental processes including root formation (Hochholdinger and Zimmermann, 2008; Peret *et al.*, 2009; Jansen *et al.*, 2012). Application of exogenous auxin or auxin transport inhibitors to roots of *Arabidopsis thaliana* and maize suggest that polar auxin transport is required for lateral root initiation (Reed *et al.*, 1998; Casimiro *et al.*, 2001; Jansen *et al.*, 2012). The semi-dominant maize mutant *rum1* (*rootless with undetectable meristem 1*) is affected in the initiation of embryonic seminal and postembryonic lateral roots of the primary root (Woll *et al.*, 2005). The mutant *rum1* showed an 83% reduction of polar auxin transport and delayed gravitropic response in the primary root (Woll *et al.*, 2005). The *rum1* gene encodes an Aux/IAA (auxin/indole-3-acetic acid) protein which is a key regulator of auxin signal transduction (von Behrens *et al.*, 2011). Canonical Aux/IAA proteins have four functional domains. Domain I is responsible for transcriptional repression (Tiwari *et al.*, 2004). In Arabidopsis, domain I is also predicted to be a protein-protein interaction domain. For instance, domain I of BD/IAA12 is responsible for interaction with TOPLESS which is required as a co-repressor for IAA12 (Szemenyei *et al.*, 2008). Domain II of Aux/IAA proteins is related to their instability of Aux/IAA proteins (Worley *et al.*, 2000; Dreher *et al.*, 2006). The degron motif GWPPV of domain II binds to the E3 ubiquitin ligase complex SCF^TIR^ (Tan *et al.*, 2007) leading to Aux/IAA protein ubiquitination and subsequent proteasomal degradation (Gray *et al.*, 2001). The maize *rum1* mutant and several Arabidopsis *Aux/IAA* gain-of-function mutants were identified due to mutations in the core region of domain II (Benjamins and Scheres, 2008; von Behrens *et al.*, 2011) resulting in increased stability of Aux/IAA proteins (Worley *et al.*, 2000; Dreher *et al.*, 2006; von Behrens *et al.*, 2011). Aux/IAA proteins can form homo- or heterodimers by their dimerization domains III and IV with Aux/IAA or ARF (auxin response factor) proteins (Kim *et al.*, 1997; Woodward and Bartel, 2005; Ludwig *et al.*, 2014). The Aux/IAA-ARF complex binds to AuxREs (auxin responsive elements) repressing the transcription of early/primary auxin-responsive genes such as *Aux/IAA*s, *SAUR*s and *GH3*s at low intracellular auxin concentrations (Woodward and Bartel, 2005). These genes often contain a conserved 5′ TGTCTC 3′ or 5′ TGTC 3′ motif in their upstream regulatory sequence (Ulmasov *et al.* 1997, 1999; Lau *et al*., 2011). However, at high cellular auxin levels, ARF proteins are released from the Aux/IAA-ARF complex to promote transcription of auxin-responsive genes, whereas Aux/IAA proteins are degraded by the proteasome (Abel, 2007; von Behrens *et al.*, 2011).

The genome of a maize progenitor was subjected to a whole genome duplication 5–12 million years ago. In the course of evolution in many instances one copy of the duplicated genes was lost, a process called partial fractionation (Schnable and Freeling, 2011). In modern maize, ~50% of all syntenic genes are pairs of homeologs while the remaining 50% of genes are single copy genes (Schnable and Freeling, 2011). Thus far only a small number of homeologous maize gene pairs have been characterized in more detail. Among those, *dwarf plant8* (*d8*) and *dwarf plant9* (*d9*) encode DELLA proteins, which are negative regulators of gibberellin signaling (Lawit *et al.*, 2010). Moreover, *colored aleurone1* (*c1*) and *purple plant1* (*pl1*) encode MYB transcription factors (Cone *et al.*, 1993), while *colored 1* (*r1*) and *colored plant* (*b1*) encode bHLH transcriptional regulators (Chandler *et al.*, 1989), which all control the biosynthesis of anthocyanin. Finally, *discordia1* (*dcd1*) and its close relative *alternative discordia1* (*add1*) encode a putative B′′ regulatory subunit of the PP2A phosphatase complex that is involved in preprophase band formation during cytokinesis (Wright *et al.*, 2009). In the present study, we characterized the unique and conserved features of the homeologous maize genes *rum1* and *rul1*, which encode Aux/IAA proteins.

## Materials and methods

### Plant material and growth conditions

Seeds of the maize inbred line B73, the mutant *rum1-R* (*rum1*-*Reference*) and its wild-type siblings were sterilized with 6% hypochlorite for 5min under vacuum at 500 mPa and then rinsed five times with distilled water. Subsequently, seeds were germinated in paper rolls in a plant growth chamber in 16h light at 28°C and 8h dark at 21°C as previously described (Ludwig *et al.*, 2013). Five-day-old seedlings of the maize inbred line B73 were treated with the auxin analog 1-Naphthaleneacetic Acid (1-NAA, working solution 5 µM) for 3h. Primary roots were harvested after 0, 1, 2 and 3h of 1-NAA exposure, then immediately frozen in liquid nitrogen and stored at −80 °C until RNA isolation (Zhang *et al.*, 2015).

### qRT-PCR expression analyses

For qRT-PCR, total RNA was isolated from distinct maize samples with the RNeasy Plant Mini Kit (Qiagen, Hilden, Germany). All RNA samples were treated with RNase-free DNaseI (Fermentas, St. Leon-Roth, Germany) and were subsequently tested for contamination with genomic DNA by PCR as previously described (Zhang *et al.*, 2015). cDNA was synthesized from 500ng total RNA using the qScript cDNA SuperMix (Quanta Biosciences, Gaithersburg, USA). qPCR was performed as previously described (Zhang *et al.*, 2014). Each genotype or treatment was assayed in four biological replicates by qRT-PCR. Each biological replicate was subjected to three qRT-PCR reactions (technical replications). An internal control gene (Genbank AC: 486090G09.x1; primers: 486090G09.x1-5′; 486090G09.x1-3′) was used in the qPCR as previously described for maize primary roots (Hoecker *et al.*, 2006). The oligonucleotide primers *rum1*-fw and *rum1*-rv, *rul1*-fw and *rul1*-rv, *rap1*-fw and *rap1*-rv were used for *rum1*, *rul1*, and *rap1* gene expression studies, respectively (Supplementary Table S1 available at *JXB* online). Differential gene expression was determined by Student’s *t*-test (*, *P*≤0.05; **, *P*≤0.01; ***, *P*≤0.001; *n*=4). Correlation of expression values was calculated based on Student’s *t*-distribution (degree of freedom, *n*−2; tails, 2).

### Subcellular localization

To construct the vector pucHA-GFP for transient transformations, pUC-SPYCE was double digested by the restriction enzymes *Sma*I and *Sac*I, then ligated with the HA-GFP fragment replacing the SPYCE fragment (Lab AC: 765). The HA-GFP open reading frame was amplified by PCR from vector pCF203-GFP using the oligonucleotide primers HA-GFP-*Sma*I-fw and HA-GFP-*Sac*I-rv (Supplementary Table S1). For the *rul1*-GFP fusion construct, the open reading frame of *rul1* which was previously cloned into the pENTR/D-TOPO vector (Invitrogen, Darmstadt, Germany) was amplified using the oligonucleotide primers *rul1*-*Kpn*I-fw and *rul1*-*Bam*HI-rv (Supplementary Table S1) introducing 5′ *Kpn*I and 3′ *Bam*HI restriction sites and deleting the stop codon of the *rul1* cDNA. Subsequently, this PCR product was introduced into the *Kpn*I and *Bam*HI restriction sites of the pucHA-GFP vector yielding a construct containing a constitutive cauliflower mosaic virus (CaMV) 35S promoter at the 5′ end of the coding sequence of *rul1* and a 3′ in-frame GFP sequence followed by the nopaline synthase (NOS) terminator (Lab AC: 818). Site-directed mutagenesis was used to change the proline (P) amino acid residues at positions 121 and 122 of RUL1 to lysine (L) according to the manufacturer’s instructions (Stratagene). The oligonucleotide primers *rul1-P121L*-fw, *rul1-P121L*-rv and *rul1-P122L*-fw, *rul1-P122L*-rv were used for site-directed mutagenesis of *rul1-P121L* and *rul1-P122L* (Lab ACs: 820 and 821), respectively (Supplementary Table S1). All of the nucleotide sequence insertions were confirmed by sequencing.

Subcellular localization experiments were performed by transiently transforming the plasmids 35S::GFP, 35S::*rul1*-GFP, 35S::*rul1-P121L*-GFP and 35S::*rul1-P122L*-GFP into Arabidopsis Col-0 protoplasts. Cell cultures were incubated at 26°C in MSCol medium overnight in dark (Liu *et al.*, 2003). Protoplasts were generated as described in Negrutiu *et al.* (1987). Transformation was performed according to a PEG protocol (Merkle *et al.*, 1996). After overnight incubation, the transformed protoplasts were examined with a HCX PL APO 63×/1.2W CORR water immersion objective (Leica Microsystems, Wetzlar, Germany) of a TCS SP2 AOBS confocal microscope (Leica Microsystems). GFP was excited with an argon laser at 488nm and the emitted fluorescence was detected with an argon-krypton laser at 509nm. Image processing was performed with Leica Confocal Software (Leica Microsystems). Epifluorescence images were taken from the same protoplasts that were analysed for green fluorescence localization.

### Stability of RUL1, rul1-P121L and rul1-P122L

The GFP constructs 35S::GFP, 35S::*rul1*-GFP, 35S::*rul1-P121L*-GFP and 35S::*rul1-P122L*-GFP that were generated for subcellular localization experiments were transformed into Arabidopsis protoplasts as described above. Sixteen hours after protoplast transformation, the auxin analog 1-NAA (working solution 10 µM) and the eukaryotic protein synthesis inhibitor cycloheximide (working solution 100 µg/ml) were added and samples were taken after 0, 10, 30, 60 and 120min. The analyses with a Modular Flow (Beckman Coulter, Brea, CA) cytometer were performed as previously described (von Behrens *et al.*, 2011). Data acquisition and analysis were performed with MoFlo Summit 4.3 software. Three biological replicates were used for each experiment.

Protein expression of the RUL1-GFP, rul1-P121L-GFP and rul1-P122L-GFP fusion protein in protoplasts was analysed by precipitating protoplasts with a buffer containing 0.5M mannitol, 15mM MgCl_2_, and 0.1% MES. Proteins were extracted using an extraction buffer containing 50mM Tris pH 7.5, 100mM NaCl, 0.1% Triton X-100 and protease inhibitor cocktail. Sixteen hours after incubation protoplasts were treated with cycloheximide and 1-NAA as described above. Per experiment, 1.5ml of the transfected protoplasts were harvested after 0, 30 and 120min. Proteins were separated on a 12% SDS-PAGE gel. Western blot analyses were performed as previously described (Saleem *et al.*, 2010) using a primary anti-GFP antibody (Roche, Germany) and a secondary anti-mouse IgG antibody (Sigma Aldrich, Germany). The secondary antibody was detected with NBT/BCIP (Roche, Germany) as previously described (Saleem *et al.*, 2010).

### Yeast two-hybrid assay

For yeast two-hybrid experiments the bait construct pGBKT7-BD-*rum1* (Lab AC: 554) was used and a cDNA expression library was generated from mRNA of 2.5-day-old maize primary roots as previously described (von Behrens *et al.*, 2011). Screening for interaction of AD-prey proteins with BD-RUM1, preparation of yeast competent cells and transformation were performed according to the manufacturer’s instructions (Clontech Laboratories, Paris, France). cDNA insertions of positive interaction partners of BD-RUM1 were sequenced and identified using http://ensembl.gramene.org.

### BiFC and flow cytometric analysis

Generation of fusion proteins of full length *rum1* and the Aux/IAA domains of ZmARF25 and ZmARF34 with YFPC (Walter *et al.*, 2004) or YFPN^152^ (Li *et al.*, 2010) has been previously described (von Behrens *et al.*, 2011). Moreover, subdomains of *rum1* were amplified with the oligonucleotide primers *DI*-*rum1*-*BamH*I-fw and *DI*-*rum1*-*Kpn*I-rv (domain I), *DII*-*rum1*-*BamH*I-fw and *DII*-*rum1*-*Kpn*I-rv (domain II) and *DIII*–*IV*-*rum1*-*BamH*I-fw and *DIII-IV*-*rum1*-*Kpn*I-rv (domain III–IV) (Supplementary Table S1) using TaKaRa La Taq Polymerase (Lonza, Basel, Switzerland) from the pENTR-TOPO-*rum1* vector (Lab AC: 473), which was constructed as previously described (von Behrens *et al.*, 2011). Subsequently, *Bam*HI and *Kpn*I fragments of domain I, domain II and domain III–IV of *rum1* were introduced into pUC-SPYCE and the modified pUC-SPYNE-152, respectively (Lab ACs: 767 and 769, 778 and 780, 768 and 770). Similarly, full length *rul1* was amplified with the oligonucleotide primers *rul1*-*Bam*HI-fw and *rul1*-*Kpn*I-rv (Supplementary Table S1) from the pENTR-TOPO-*rul1* (Lab AC: 475) vector, which was generated as described above. Subsequently, *Bam*HI and *Kpn*I fragments were introduced into pUC-SPYCE and the modified pUC-SPYNE-152 (Lab ACs: 529 and 530). Furthermore, the oligonucleotide primers *rum1*-*R*-*Xba*I-fw and *rum1*-*R*-*Sma*I-rv (Supplementary Table S1) were used to amplify the full length sequence of mutated *rum1*-*R* cDNA using pENTR-TOPO-*rum1-R* as a template (Lab AC: 474) (von Behrens *et al.*, 2011). This PCR product was introduced into the restriction sites *Xba*I and *Sma*I of pUC-SPYCE and the modified pUC-SPYNE-152 vectors (Lab ACs: 748 and 749). Finally, full length *rap1* was generated by PCR amplification with *rap1*-fw and *rap1*-rv oligonucleotide primers (Supplementary Table S1). Subsequently, this template was reamplified with the oligonucleotide primers *rap1*-*Xba*I-fw and *rap1*-*Sma*I-rv (Supplementary Table S1) to introduce *Xba*I and *Sma*I restriction sites, which were then introduced into the restriction sites of the BiFC vector pUC-SPYCE and the modified vector pUC-SPYNE-152 (Lab ACs: 710 and 711). The inserts were confirmed by sequencing and transformed into Arabidopsis Col-0 protoplasts for BiFC analyses.

Flow cytometry was performed as previously described (von Behrens *et al.*, 2011). Briefly, 300 µl of the transfected protoplasts per experiment were filtered through a 40 μM sieve and fluorescence signal intensity was analysed with a Modular Flow (Beckman Coulter, Brea, CA, USA) cytometer. YFP fluorescence was excited with a 488nm (50 mW) argon laser and its principal emission was captured in FL1 (530/40) and plotted against autofluorescence in FL2 (580/30). After gating out cellular debris detected in the FSC/SSC plot, BiFC expressing cells were identified as those whose fluorescence was increased in the FL1 channel compared to the negative controls (NUO-YFPCE and NUO-YFPNE-152, Lab ACs: 705 and 706) as previously described (von Behrens *et al.*, 2011). Data acquisition and analysis was performed with MoFlo Summit 4.3 software.

Protein expression of each fusion protein described above was analysed in Arabidopsis protoplasts. Western blot analyses were performed as previously described (Saleem *et al.*, 2010) using a primary anti-HA antibody (Roche, Germany) or anti-MYC antibody (Cell Signaling Technology, USA) and a secondary anti-mouse IgG antibody (Sigma Aldrich, Germany). The secondary antibody was detected with NBT/BCIP (Roche, Germany) as described before (Saleem *et al.*, 2010).

## Results

### RUL1 is the homeolog of the Aux/IAA protein RUM1 and localizes to the nucleus

**Fig. 1.**
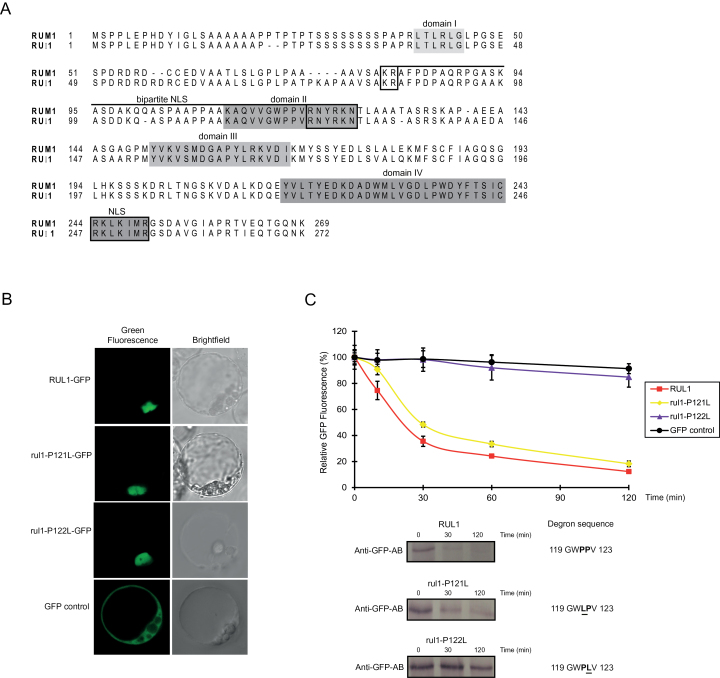
Characteristics of *rul1*. (A) Alignment of the amino acid sequences encoded by the homeologous genes *rul1* and *rum1* revealed ~92% identity. The domain structures and the nuclear localization signal (NLS) are indicated. (B) Subcellular localization of RUL1, rul1-P121L-GFP and rul1-P122L-GFP and a GFP control. (C) Protein stability assay of RUL1, rul1-P121L, rul1-P122L and the GFP control. Relative fluorescence of these proteins was measured in Arabidopsis Col-0 protoplasts between 0 and 120min after 1-NAA and cycloheximide treatment by flow cytometry. Protein stability of RUL1-GFP, rul1-P121L-GFP and rul1-P122L-GFP was confirmed at time points 0, 30, 120min by Western blot analyses using an anti-GFP antibody.

Based on their microsynteny it has been demonstrated that *rum1*, which is located on chromosome 3, and *rul1* (*rum1*-*like1*; GRMZM2G163848), which maps to chromosome 8, are homeologs (von Behrens *et al.*, 2011). A maize progenitor underwent a whole genome duplication in ancient times. Therefore, many genomic regions of modern maize can be attributed to either of two subgenomes 1 and 2. While the *rum1* gene belongs to subgenome 1 its homeolog *rul1* was attributed to subgenome 2. RUL1 is predicted to contain the canonical four domain structure of Aux/IAA proteins and a bipartite nuclear localization signal (NLS; Fig. 1A). To determine the subcellular localization of RUL1, a RUL1-GFP fusion protein was transiently expressed in Arabidopsis Col-0 protoplasts. This experiment localized RUL1-GFP to the nucleus (Fig. 1B). Similarly, the fusion proteins rul1-P121L-GFP and rul1-P122L-GFP containing point mutations in the degron sequence of RUL1 were also localized to the nucleus. By contrast, the GFP control protein displayed a constitutive localization in the nucleus and the cytoplasm.

### RUL1 is unstable

Aux/IAA proteins are unstable proteins that are rapidly degraded at increased cellular auxin levels. Therefore, the stability of the RUL1 wild-type protein was compared with two mutated isoforms of the protein, rul1-P121L and rul1-P122L in which the proline (P) at amino acid positions 121 and 122 was changed into lysine (L), respectively. Relative GFP-fluorescence intensities of RUL1-GFP, rul1-P121L-GFP and rul1-P122L-GFP fusion proteins were measured between 0 and 120min with a flow cytometer (Fig. 1C). As a control, relative GFP-fluorescence intensities of constitutively expressed GFP were determined (Fig. 1C). This experiment demonstrated the instability of RUL1 with an average half-life of ~23min, compared to the stable GFP protein (Fig. 1C). As predicted, the P-to-L amino acid exchange within the degron sequence of RUL1 at position 122 stabilized the protein significantly. While the relative GFP fluorescence of wild-type RUL1 was reduced to ~10% within 120min, GFP fluorescence of rul1-P122L was still at ~85% which is close to the GFP control. By contrast, the P-to-L amino acid exchange within the degron sequence of RUL1 at position 121 did not stabilize the protein. The rul1-P121L protein displayed a similar half-life of ~28min as the wild-type RUL1 protein and after 120min only ~20% of the GFP fluorescence was remaining (Fig. 1C). Western blot experiments with total protein extracts from protoplasts overexpressing RUL1-GFP, rul1-P121L-GFP and rul1-P122L-GFP fusion proteins confirmed these results by showing half-lives similar in value to those observed in flow cytometry (Fig. 1C).

### Overall higher expression in *rul1* compared to *rum1*

**Fig. 2.**
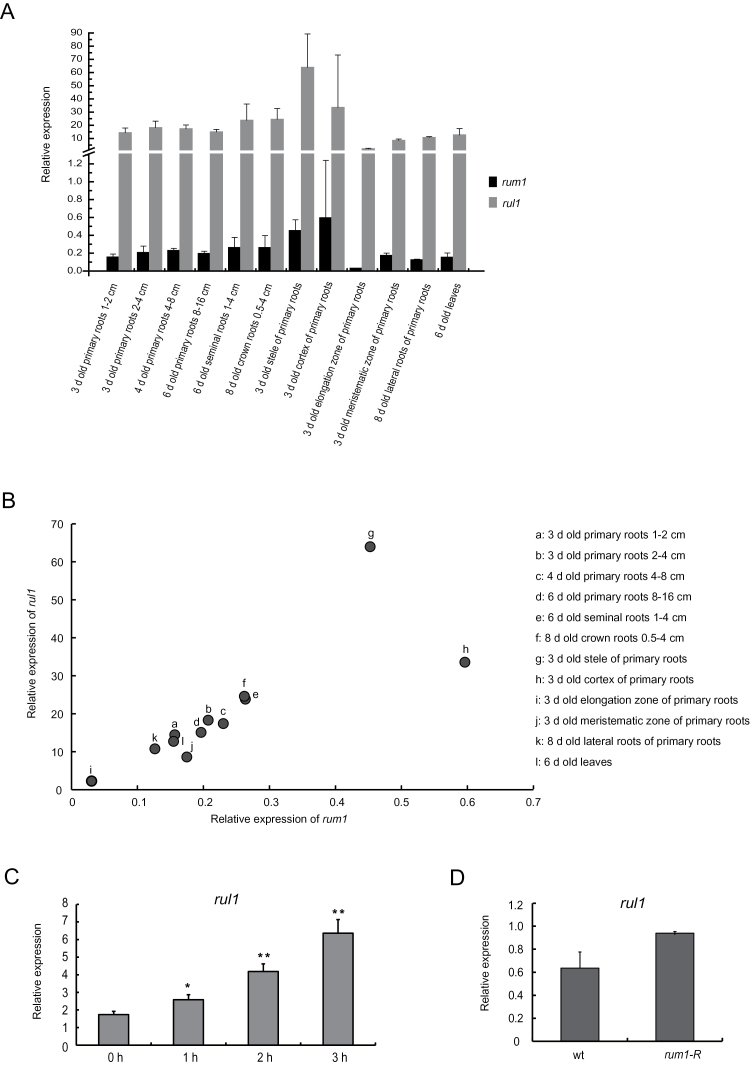
Expression of *rum1* and *rul1*. (A) qRT-PCR analyses of *rul1* versus *rum1* in 12 different tissues at different developmental stages. (B) Correlation of *rum1* and *rul1* gene expression patterns. (C) Auxin induction of *rul1* in primary roots of wild-type seedlings assayed by qRT-PCR after treatment with 5 µM 1-NAA at 0, 1, 2 and 3h, respectively (*, *P*≤0.05; **, *P*≤0.01; *n*=4). (D) Expression of *rul1* in wild-type versus *rum1*-*R* primary roots assayed by qRT-PCR experiments (Fc=1.5; *P*≤0.1).

To compare the temporal and spatial expression patterns of *rum1* and *rul1*, 12 distinct tissues and developmental stages of the maize inbred line B73 were analysed by qRT-PCR (Fig. 2A). In all comparisons, *rul1* was significantly higher expressed than *rum1*. On average, *rul1* displayed a ~84-fold higher expression than *rum1*. Both, *rum1* and *rul1* displayed the highest expression in the cortex and stele of 3-day-old primary roots (Fig. 2A). Among all tested tissues, *rum1* and *rul1* displayed the lowest expression in the elongation zone of 3-day-old primary roots. Furthermore, both genes were expressed at higher levels in seminal and crown roots than in all other tested stages of primary roots. Despite the significant differences in overall expression, across all tissues *rum1* and *rul1* expression was significantly correlated (Fig. 2B). For all analysed tissues a Pearson correlation coefficient of R=0.8 (*P*≤0.01) was calculated. Pairwise *t*-tests comparing expression of the two genes in all tested tissues are summarized in Supplementary Table S2. Thus far two types of auxin response promoter elements (AuxRE), 5′ TGTCTC 3′ and 5′ TGTC 3′, have been described. Promoter analysis of 1kb upstream of the ATG start codon of *rul1* revealed six 5′ TGTC 3′ elements. Auxin-inducibility of *rul1* was tested by qRT-PCR in 5-day-old B73 primary roots after 5 µM 1-NAA treatment (Fig. 2C). The experiment demonstrated that *rul1* is auxin inducible (FC=3.7) within 3h. Only a relatively small expression difference of *rul1* at a moderate significance was observed in wild-type vs. *rum-R* mutant primary roots (Fig. 2D), suggesting that *rul1* expression might not be controlled by *rum1*.

### RUL1 interacts with ZmARF25 and ZmARF34, RUM1 and itself

Aux/IAA proteins are characterized by their capability of interacting with ARF proteins. Interactions of RUL1 with ZmARF25 and ZmARF34, which have been previously demonstrated to interact with RUM1 (von Behrens *et al.*, 2011), were surveyed in BiFC (Bimolecular Fluorescence Complementation) experiments. This *in vivo* technique is based on the detection of a YFP (Yellow Fluorescent Protein) signal which is emitted when N (YFPN) and C (YFPC) terminal YFP parts come in close proximity by the interactions of fusion proteins coupled to these YFP subunits.

**Fig. 3.**
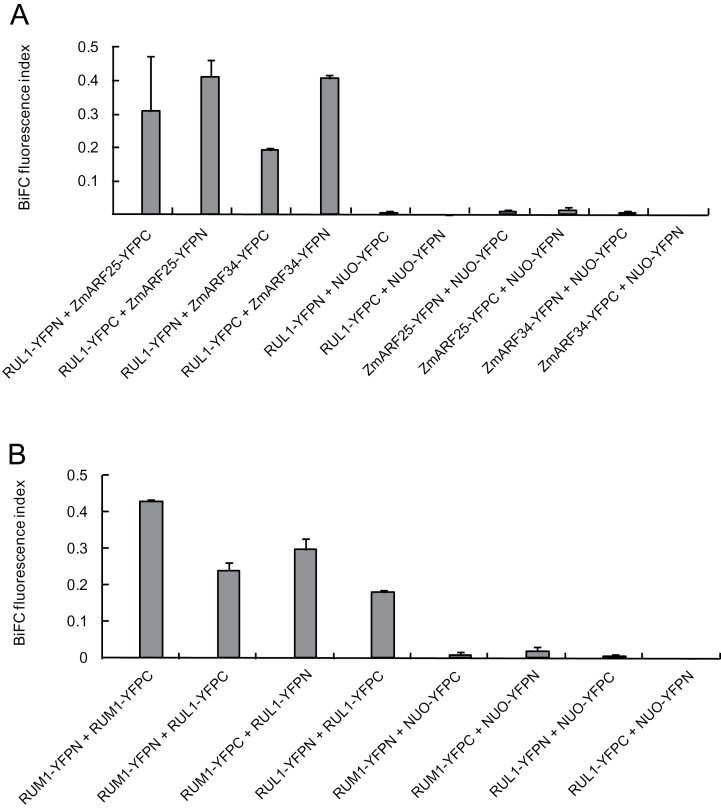
Quantification of RUL1 protein interactions with ZmARF25, ZmARF34 and RUM1 by the BiFC system in Arabidopsis Col-0 protoplasts. (A) RUL1 interaction with ZmARF25 and ZmARF34. (B) Homo- and hetero-interaction of RUM1 and RUL1.

Quantification of the YFP fluorescence by flow cytometry demonstrated significant interactions of RUL1 with ZmARF25 and ZmARF34 in Arabidopsis protoplasts compared to the control experiments (Fig. 3A). Moreover, significant homo-interaction was observed for RUM1-RUM1 and RUL1-RUL1. Furthermore, hetero-interaction of RUM1-RUL1 in both orientations in comparison to the corresponding negative controls was demonstrated (Fig. 3B). Each experiment was performed in three biological replicates. The fusion proteins NUO-YFPC and NUO-YFPN, which were used as a negative control (von Behrens *et al.*, 2011), did not display any interaction with RUL1, ARF25, and ARF34. Expression of fusion proteins in BiFC assays according to Fig. 3A, B were confirmed by Western blot experiments (Supplementary Fig. S1).

### RAP1specifically interacts with RUM1 but not with RUL1

**Fig. 4.**
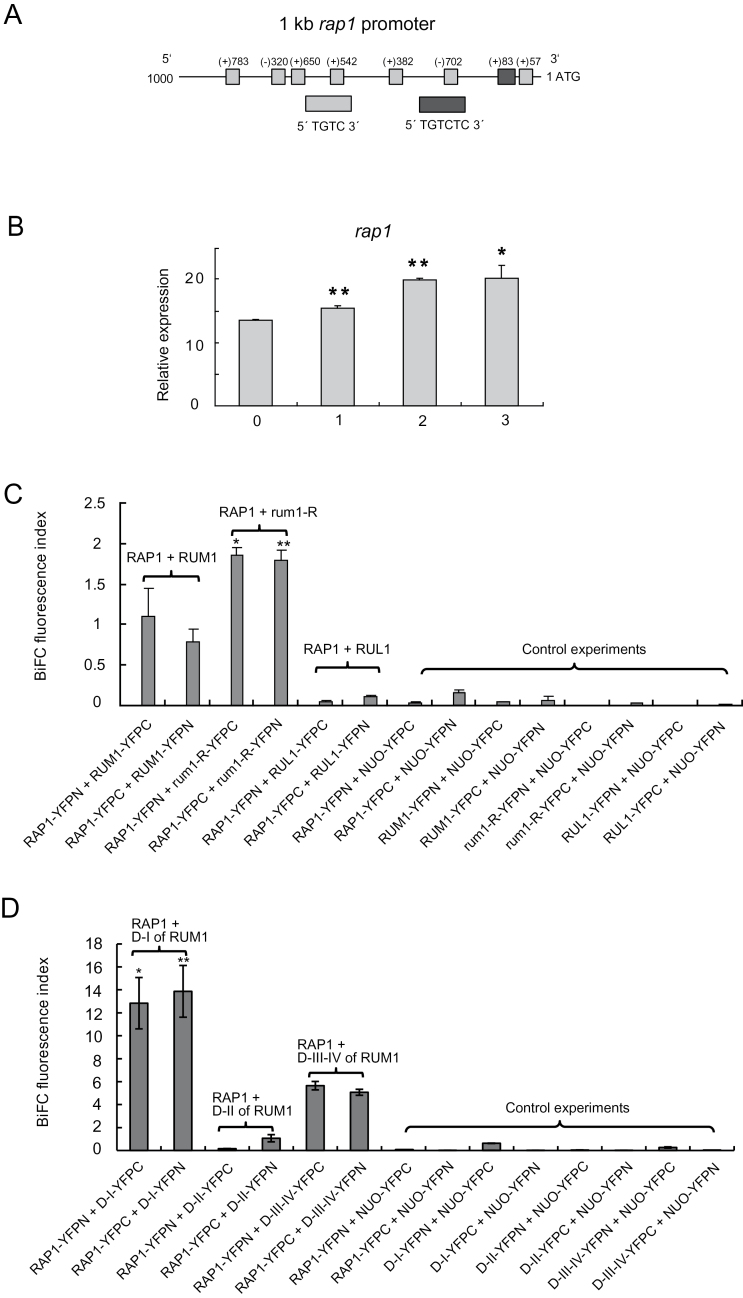
Auxin related characteristics of *rap1* and specific interaction of RAP1 with RUM1 but not RUL1. (A) AuxRE analysis of 1-kb *rap1* promoter. 5′ TGTCTC 3′ is represented by a dark grey box. 5′ TGTC 3′ is denoted by a light grey box. (B) Expression of *rap1* gene in primary roots of wild-type seedlings after treatment with 5 µM 1-NAA at 0, 1, 2 and 3h, respectively, assayed by qRT-PCR (*, *P*≤0.05; **, *P*≤0.01; n=3). (C) RAP1 interaction with RUM1, rum1-R and RUL1. (D) RAP1 interaction with domains I, II and III–IV of RUM1. In all experiments the NUO protein was used as a negative control.

To identify novel interaction partners for RUM1 and RUL1, a yeast two-hybrid assay was performed using RUM1 as bait and a cDNA expression library generated from mRNA of 2.5-day-old maize primary roots as prey (methods). This experiment revealed known interaction partners of RUM1 and RUL1 such as RUM1 and ARF25 (Supplementary Table S3). To identify interaction partners of RUM1 and RUL1 involved in auxin signal transduction, 1kb promoter sequences upstream of the ATG start codon of yeast two-hybrid candidate genes were screened for auxin response elements (AuxREs). The promoter of a gene designated *rap1* contained seven AuxREs of the type 5′ TGTCTC 3′ (once) and 5′ TGTC 3′ (six times) (Fig. 4A). Auxin induction of *rap1* in 5-day-old wild-type primary roots was demonstrated after a 3h treatment with 5 µM 1-NAA (Fig. 4B).

Subseqeunetly, BiFC experiments were performed to analyse the interaction of RAP1 (RUM1 ASSOCIATED PROTEIN 1) with RUM1, RUL1 and rum1-R, a mutated form of RUM1 lacking 24 amino acids in domain II. These experiments confirmed the interaction of RUM1 and rum1-R with RAP1 in Arabidopsis protoplasts. Remarkably, interaction of the mutant protein rum1-R with RAP1 in Arabidopsis protoplasts was significantly stronger than RUM1-RAP1 interaction (Fig. 4C), possibly due to the increased stability of the mutated proteins. In contrast, no interaction was observed between RUL1 and RAP1 compared to the corresponding negative controls in BiFC analyses (Fig. 4C). Western blot analyses were performed with Anti-HA (haemagglutinin) antibodies against the YFPC and anti-c-Myc antibodies against the YFPN tag, which demonstrated the expression of the corresponding proteins in Arabidopsis protoplasts (Supplementary Fig. S1).

BiFC assays with domain I, II, and III-IV of RUM1 with RAP1 were performed to resolve which domain of the RUM1 protein interacts with RAP1. These experiments demonstrated that the interaction of domain I of RUM1 with RAP1 was significantly stronger than the interaction of domain III-IV of RUM1 with RAP1 (Fig. 4D). In contrast, no interaction was detected between domain II of RUM1 and RAP1 in BiFC experiments relative to the negative controls (Fig. 4D). These results suggested that domain I of RUM1 is mainly responsible for interaction with RAP1 although the truncated domain III-IV in RUM1 also interacted with RAP1. Expression of all experimental fusion-proteins in Arabidopsis protoplasts were confirmed by Western blot assays (Supplementary Fig. S1).

### The RAP1 family in maize

Homology searches using the maize RAP1 protein sequence as query revealed six additional proteins of this family ZmRAP1-like1 (ZmRAL1) to ZmRAP1-like6 (ZmRAL6). Four of seven maize genes were assigned to maize subgenome 1, while the remaining three members were not assigned to a subgenome. All four maize genes assigned to subgenome 1 (ZmRAL1, ZmRAL3, ZmRAL5, ZmRAL6) have an ortholog in rice and sorghum (Supplementary Table S4).

## Discussion

### RUM1 and RUL1 display characteristics of canonical Aux/IAA proteins

About 5–12 million years ago a maize progenitor had undergone a whole genome duplication which led to the emergence of two subgenomes (Schnable *et al.*, 2011). During evolution, subgenome 2 experienced more gene loss than subgenome 1. Moreover, mutant phenotypes identified in forward genetic screens are often the result of a mutation in genes of maize subgenome 1 (Schnable and Freeling, 2011). This observation is explained by the hypothesis that subgenome 1 genes have predominantly retained the ancestral function while subgenome 2 genes potentially adopted new, or less essential functions (Schnable and Freeling, 2011). In line with this, several genes controlling maize root development such as *rtcs* (Taramino *et al.*, 2007), *rth3* (Hochholdinger *et al.*, 2008) and *rum1* (von Behrens *et al.*, 2011) belong to subgenome 1. Their homeologs in maize subgenome 2 are designated *rtcl* (Taramino *et al.*, 2007), *rtl3* (Hochholdinger *et al.*, 2008) and *rul1* (von Behrens *et al.*, 2011).

The proteins encoded by *rum1* and *rul1* display the canonical four domain architecture defining Aux/IAA proteins (Liscum and Reed, 2002). Aux/IAA proteins are involved in the transcriptional regulation of auxin responsive genes (Quint and Gray, 2006) and are therefore localized in the nucleus. In the present study, nuclear localization of RUL1 was demonstrated by RUL1-GFP localization in Arabidopsis protoplasts (Fig. 1B). This localization pattern was also observed for RUM1 (von Behrens *et al.*, 2011) and several other Aux/IAA proteins (Ludwig *et al.*, 2014).

Aux/IAA proteins are unstable with short half-lives of between six and 80 minutes due to their interaction with the SCF^TIR^ complex via domain II and subsequent proteasomal degradation (Abel *et al.*, 1994; Gray *et al.*, 2001; Ouellet *et al.*, 2001). The instability of Aux/IAA proteins is conferred by interaction with the SCF^TIR1^ complex at the conserved degron sequence GWPPV (Dreher *et al.*, 2006). Point mutations in this short amino acid stretch are often sufficient to prohibit the interaction and thus enhance the stability of Aux/IAA proteins (Tian *et al.*, 2003). In the present study, wild-type RUL1-GFP displayed a half-life of ~23min (Fig. 1C) which was similar to RUM1 (~22min; von Behrens *et al.*, 2011). Remarkably, while the point mutation that led to a P-to-L exchange in position 122 was sufficient to stabilize the rul1-P122L protein, the same amino exchange at position 121 did not stabilize the protein (Fig. 1C) suggesting that SCF^TIR1^ interaction and subsequent proteasomal degradation is still possible in this mutated protein to a considerable degree. In Arabidopsis several mutants with a developmental phenotype have been identified with P-to-L exchanges that correspond to position 121 (Rogg *et al.*, 2001; Tatematsu *et al.*, 2004) and 122 (Rouse *et al.*, 1998; Knox *et al.*, 2003; Tatematsu *et al.*, 2004) in the degron sequence. These aberrant phenotypes are likely conditioned by stabilized mutated Aux/IAA proteins. In maize, as suggested by rul1-P121L-GFP and rul1-P122L-GFP, distinct positions in the degron motif might contribute differently to protein interaction with the SCF^TIR^ complex.

Aux/IAA proteins repress the transcription of early auxin-responsive genes by their interaction with ARF proteins (Woodward and Bartel, 2005). In the present study, we demonstrated interaction of RUL1 with ZmARF25 and ZmARF34 (Fig. 3A) as previously demonstrated for RUM1 (von Behrens *et al.*, 2011). It was suggested that this interaction blocks lateral root formation in non-precursor pericycle cells (von Behrens *et al.*, 2011). In Arabidopsis, multiple models of Aux/IAA-ARF-dependent auxin response signalling involved in lateral root development were proposed. First, the IAA28-ARF-dependent model mediates the specification of lateral root founder cell identity (De Rybel *et al.*, 2010). Second, the SLR/IAA14-ARF7-ARF19 module controls the division of early founder cells of lateral roots, and subsequently the BDL/IAA12-ARF5 module regulates lateral root initiation and organogenesis (De Smet *et al.*, 2010). Hence, RUM1 and RUL1 might also be involved in different pathways involved in lateral root formation.

In addition to the canonical features of RUL1, it was demonstrated in the present survey that RUM1 and RUL1 can form homo- and heterodimers *in vivo* (Fig. 3B). It was previously demonstrated that the Arabidopsis proteins IAA1, IAA2 and the pea protein IAA4 can form homodimers *in vitro* (Kim *et al.*, 1997). Nevertheless, the function of Aux/IAA interactions still remains elusive and it has been suggested that it might allow interaction with downstream genes without the formation of Aux/IAA-ARF complexes (Paciorek and Friml, 2006). Similarly, direct binding of RUM1 to the promoter of *lrp1* (*lateral root primordia 1*) has also been suggested (Zhang *et al.*, 2015).

### In general *rul1* displays higher expression than *rum1*

It was observed that on average genes of maize subgenome 1 were expressed at higher levels than their homeologous genes in subgenome 2 (Schnable *et al.*, 2011). In contrast to this trend, *rul1* (subgenome 2) displayed on average a ~84-fold higher expression than *rum1* (subgenome 1) in the 12 tissues surveyed in the present study (Fig. 2A). Despite highly correlated expression patterns (Fig. 2B), the significantly differential expression intensities of *rul1* and *rum1* might suggest distinct functions of these two homeologous proteins in root development.

Promoter analysis of the sequence 1kb upstream of the ATG start codon revealed 13 putative AuxREs in *rum1* and six AuxRE in *rul1*. Despite the different number and position of AuxRE elements it was demonstrated that *rul1* is auxin inducible as previously demonstrated for *rum1* (von Behrens *et al.*, 2011). Moreover, by comparing *rul1* expression in wild-type and *rum1-R* mutant primary roots it was demonstrated that the expression of *rul1* was not regulated by *rum1*. This result supports the notion that these genes might act in different molecular pathways and might therefore have different functions in root development.

For the recessive loss-of-function mutants *rtcs* and *rth3*, different functions compared to their homeologous genes were demonstrated since the homeologs were not able to complement the mutant phenotypes (Taramino *et al.*, 2007; Hochholdinger *et al.*, 2008). However for the semi-dominant mutation *rum1-R* the situation is different. The *rum1-R* mutant phenotype is conferred by a stabilization of the rum1-R/ARF complex, which inhibits the expression of downstream gene expression (von Behrens *et al.*, 2011). Hence, RUL1 cannot complement the mutant phenotype because downstream gene expression is already blocked by the gene product of the gain-of-function allele rum1-R which may not allow redundancy in this process.

### RAP1 specifically interacts with RUM1 but not with RUL1

AtSPR1 is a plant-specific small protein. Homology searches revealed similarity of AtSPR1 with a nitrilase-associated protein (GenBank AC: Z96936) in Arabidopsis (Nakajima *et al.*, 2004). Yeast two-hybrid experiments demonstrated interaction of RUM1 with a novel protein which is a homolog of AtSPR1 and which we designated RAP1. Homology searches (ensembl.gramene.org) identified a total of seven homeologous maize genes *rap1* and *rap1-like1* (*ral1*) to *ral6* (Supplementary Table S4). Four of seven *rap1-like* gene family members (57%) were assigned to maize subgenome 1 (Supplementary Table S4). This tendency was also observed for the *lrp1-like* gene family where five of nine (56%) assigned to subgenome 1. The remaining three (43%) *rap1-like* genes likely emerged by single copy duplications after the last whole genome duplication because they did not map to any of the subgenomes. Similarly, seven *Aux/IAA* genes were not associated with a subgenome, suggesting that they emerged after the ancient genome duplication of maize (Ludwig *et al.*, 2013).

Quantification of the interaction of RUM1, rum1-R and RUL1 with RAP1 (Fig. 4C) demonstrated interaction of RUM1 with RAP1, and an even stronger interaction of rum1-R with RAP1 *in vivo*. This is most likely a consequence of the observation that rum1-R is more stable than RUM1 (von Behrens *et al.*, 2011). The homeologous maize proteins RUM1 and RUL1 share 92% identity on the protein level (von Behrens *et al.*, 2011). However, no interaction was detected between RUL1 and RAP1. A domain interaction analysis revealed that the interaction of domain I of RUM1 with RAP1 is significantly stronger than the interaction of domains III–IV, while no interaction was detected with domain II in BiFC experiments (Fig. 4D). It has been demonstrated that domain I of Aux/IAA is also responsible for protein-protein heterodimerization. For example, domain I of BDL/IAA12 was sufficient to interact with TOPLESS which is a co-repressor regulating embryogenesis in Arabidopsis (Szemenyei *et al.*, 2008).

In summary, we demonstrated that both RUM1 and RUL1 display all characteristics of functional Aux/IAA proteins. Distinct functions of the two proteins are suggested by a different promoter architecture and overall differences in gene expression levels. Moreover, it was demonstrated that *rul1* is not regulated by RUM1 suggesting their activity in independent pathways. Finally, RUM1 specific interaction with RAP1 suggests that these homeologous genes, despite their role as Aux/IAA proteins, have at least in part diverse interaction partners and might thus be functioning in distinct molecular networks.

## Supplementary data

Supplementary data are available at *JXB* online.

Fig. S1. Expression of fusion proteins in Arabidopsis Col-0 protoplasts detected by Western blot experiments.

Table S1. Sequences of oligonucleotide primers used in this study.

Table S2. Pairwise comparison of *rum1* (upper table) and *rul1* (lower table) expression in different tissues and at different developmental stages according to Fig. 2A.

Table S3. RUM1 interaction partners identified via yeast two-hybrid experiments.

Table S4. Characteristics of members of the maize *rap1-like* gene family in maize.

Supplementary Data
