# Supplementary material for: Conserved and unique features of the homeologous maize Aux/IAA proteins ROOTLESS WITH UNDETECTABLE MERISTEM 1 and RUM1-like 1
Source: J Exp Bot. 2015 Dec 15;67(4):1137–47. doi: 10.1093/jxb/erv519 (PMC4753850; doi:10.1093/jxb/erv519)
Supplement: Supplementary Data [file supp_67_4_1137__index.html]

Conserved and unique features of the homeologous maize Aux/IAA proteins ROOTLESS WITH UNDETECTABLE MERISTEM 1 and RUM1-like 1 — Conserved and unique features of the homeologous maize Aux/IAA proteins ROOTLESS WITH UNDETECTABLE MERISTEM 1 and RUM1-like 1 — Supplementary Data 

# Conserved and unique features of the homeologous maize Aux/IAA proteins ROOTLESS WITH UNDETECTABLE MERISTEM 1 and RUM1-like 1

## Supplementary Data

Data files

- supplementary\_figure\_S1\_tables\_S1\_S4.pdf - Supplementary Data
