# Supplementary material for: Conserved and unique features of the homeologous maize Aux/IAA proteins ROOTLESS WITH UNDETECTABLE MERISTEM 1 and RUM1-like 1
Source: J Exp Bot. 2015 Dec 15;67(4):1137–47. doi: 10.1093/jxb/erv519 (PMC4753850; doi:10.1093/jxb/erv519)
Supplement: Supplementary Data [file supp_erv519_supplementary_figure_S1_tables_S1_S4.pdf]

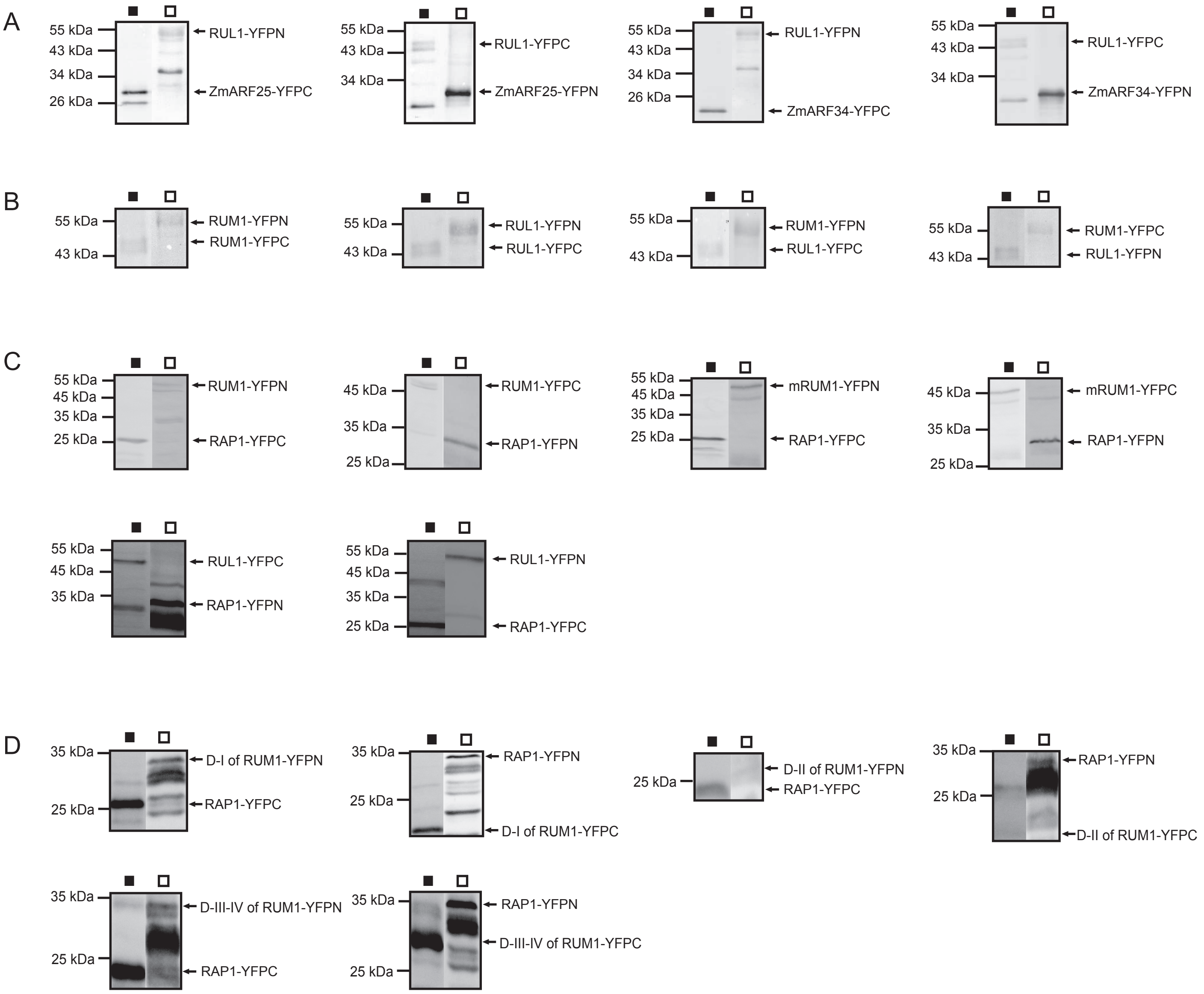

Supplementary Fig. S1. Expression of fusion proteins in Arabidopsis Col-0 protoplasts detected by Western blot experiments. YFPC fusion proteins were detected with anti-HA-antibodies indicated by black squares, YFPN fusion proteins were detected by anti-c-Myc-antibodies indicated by white squares. (A) Interaction of RUL1 with ZmARF25 and ZmARF34. (B) Homo and heterodimerization of RUM1 and RUL1. (C) Interaction of RUM1, RUL1 and mutant rum1-R with RAP1. (D) Interaction of domain I of RUM1 with RAP1, domain II of RUM1 with RAP1, and domain III-IV of RUM1 with RAP1.

Supplementary Table S1. Sequences of oligonucleotide primers used in this study.

qRT-PCR:

**Name**	**Sequence**
Fw-primer (*rap1*-fw)	5´ ACACTGGCAACTTCCTTACG 3´
Rv-primer (*rap1*-rv)	5´ CGGTTGTTCACGATTCAGAC 3´
Fw-primer (*rum1*-fw)	5´ CCTGCATCCAAGGAAGACAT 3´
Rv-primer (*rum1*-rv)	5´ CTTGACATCACGAACCATCG 3´
Fw-primer (*rul1*-fw)	5´ CTCCAAGAACCATAGAGCAG 3´
Rv-primer (*rul1*-fw)	5´ CGTCTCAAGAACACGTGGAA 3´
Fw-primer (AY104722-fw-210)	5´ ATGTGACAATGGCACTGGAA 3´
Rv-primer (AY104722-rv-925)	5´ GACCTGACCATCAGGCATCT 3´
Fw-primer (486090G09.x1-5´)	5´ CAAGGAGAGACTCTGTGAGCTTCA 3´
Rv-primer (486090G09.x1-3´)	5´ AGAAGGCCGTACAGGATCTTACC 3´

Subcellular localization and stability of RUL1 (restriction sites underlined):

**Name**	**Sequence**
Fw-primer (*HA-Gfp-Sma*I-fw)	5´ CCCCCGGGATGTACCCATACGATGTTCCAGATTACGCTATGAGTAAAGGAGAAGAACTT 3´
Rv-primer (*HA-Gfp-Sac*I-rv)	5´ GCGAGCTCTTATTTGTATAGTTCATCCAT 3´
Fw-primer (*rul1-Kpn*I-fw)	5´ ACACACAAGGTACCATGGCGCCGCC 3´
Rv-primer (*rul1-Bam*HI-rv)	5´ ACACTCCCGGATCCATTTGTTCTGACCTGTCTG 3´

Site-directed mutagenesis of RUL1:

**Name**	**Sequence**
Fw-primer (*rul1-P121L*-fw)	5´ GTGGTGGGATGGCTGCCCGTGCGGAACTAC 3´
Rv-primer (*rul1-P121L*-rv)	5´ GTAGTTCCGCACGGGCAGCCATCCCACCAC 3´
Fw-primer (*rul1-P122L*-fw)	5´ GTGGGATGGCCGCTCGTGCGGAACTACC 3´
Rv-primer (*rul1-P122L*-rv)	5´ GGTAGTTCCGCACGAGCGGCCATCCCAC 3´

BiFC and FACS (restriction sites underlined):

**Name**	**Sequence**
Fw-primer (*DI-rum1-Bam*HI-fw)	5´ ACACACAAGGATCCATGACCTCCTCCTCCTCTTCGTC 3´
Rv-primer (*DI-rum1-Kpn*I-rv)	5´ AATGGGGTACCGTCGCTAGCCTTGGAAGC 3´
Fw-primer (*DII-rum1-Bam*HI-fw)	5´ ACACACAAGGATCCATGGGCGCTTCCAAGGCTAGCGA 3´
Rv-primer (*DII-rum1-Kpn*I-rv)	5´ AATGGGGTACCCTTGCTCCTGGAGGCGGTCG 3´
Fw-primer (*DIII-IV-rum1-Bam*HI-fw)	5´ ACACACAAGGATCCATGTACGTGAAGGTGAGCAT 3´

Rv-primer (*DIII-IV-rum1-Kpn*I-rv)	5´ ACACACACGGTACCTTTGTTCTGACCTGTCTG 3´
Fw-primer (*rap1*-fw)	5´ GTGCTTGATACCTGATTAGA 3´
Rv-primer (*rap1*-rv)	5´ GATGAAGGTTCAGGCACACG 3´
Fw-primer (*rap1-Xba*I-fw)	5´ GAGCTCTAGAATGAGCCGTGGTGGTAGT 3´
Rv-primer (*rap1-Sma*I-rv)	5´ CAAAATCCCCCGGGGTTGCCACCGAACAGGTA 3´
Fw-primer (*rul1-Bam*HI-fw)	5´ AAAAACAAGGATCCATGGCGCCGCC 3´
Rv-primer (*rul1-Kpn*I-rv)	5´ ACACACACGGTACCTTTGTTCTGACCTGTCTG 3´
Fw-primer (*rum1-R-Xba*I-fw)	5´ GCTCTAGAATGGCGCCGCCCCTCGAGCC 3´
Rv-primer (*rum1-R-Sma*I-rv)	5´ TCCCCCGGGTTTGTTCTGACCTGTCTGC 3´
Fw-primer (*rum1-Bam*HI-fw)	5´ AAAAACAAGGATCCATGGCGCCGCC 3´
Rv-primer (*rum1-Kpn*I-rv)	5´ ACACACACGGTACCTTTGTTCTGACCTGTCTG 3´
Fw-primer (*ZmARF25-Xba*I-fw)	5´ AGCGGCGCTCTAGAATGACATTTACCAAGGTGTAC 3´
Rv-primer (*ZmARF25-Xho*I-rv)	5´ CCACAAAACTCGAGCTGAGGCGACAGGATCC 3´
Fw-primer (*ZmARF34-Xba*I-fw)	5´ ACCCCCACTCTAGAATGACCTTCACCAAGGTTC 3´
Rv-primer (*ZmARF34-Xho*I-rv)	5´ CCACCCCCCTCGAGAACAGATGACAATATTTTAATGC 3´
Fw-primer (*nuo-Xba*I-fw)	5´ GCTCTAGAATGGCGTCGACGGCTGGGTA 3´
Rv-primer (*nuo-Sma*I-rv)	5´ TCCCCCGGGCTGGTCCTCCTTGCCATAAT 3´

Supplementary Table S2. Pairwise comparison of *rum1* (upper table) and *rul1* (lower table) expression in different tissues and at different developmental stages according to Fig. 2A. Differential gene expression was determined by Student´s t-test (*: $p \leq 0.05$; **: $p \leq 0.01$; ***: $p \leq 0.001$).

rum1	Primary roots 1-2cm	Primary roots 2-4cm	Primary roots4-8cm	Primary roots 8-16cm	Seminal roots 1-4cm	Crown roots 0,5-4cm	Stele	Cortex	Elongation zone	Meristematic zone	Lateral roots	Leaves
Primary roots 1-2cm			***		*		**		**			
Primary roots 2-4cm							*		**			
Primary roots 4-8cm							*		***	*	***	*
Primary roots 8-16cm							*		***		**	
Seminal roots 1-4cm							*		*		*	
Crown roots 0,5-4cm									*			
Stele									**	***	**	**
Cortex										*		
Elongation zone										***	*	**
Meristematic zone											*	
Lateral roots												
Leaves												

rul1	Primary roots 1-2cm	Primary roots2-4cm	Primary roots 4-8cm	Primary roots 8-16cm	Seminal roots 1-4cm	Crown roots 0,5-4cm	Stele	Cortex	Elongation zone	Meristematic zone	Lateral roots	Leaves
Primary roots 1-2cm						*	*		**	*		
Primary roots 2-4cm						*	*		**	*	*	
Primary roots 4-8cm							*		***	**	**	*
Primary roots 8-16cm							*		***	**	**	
Seminal roots 1-4cm							*		*			
Crown roots 0,5-4cm							*		**	*	*	*
Stele									**	*	*	*
Cortex												
Elongation zone										***	**	**
Meristematic zone											*	
Lateral roots												
Leaves												

Supplementary Table S3: RUM1 interaction partners identified via yeast-two-hybrid experiments.

Protein identification	Ensembl.gramene.org accession No.	No. of positive clones
Auxin Response Factor 25[a]	GRMZM2G317900_P01	1
RUM1 [a]	GRMZM2G037368_P01	1
RAP1 (RUM1 ASSOCIATED PROTEIN 1) similar to AtSPR1 [a]	GRMZM2G124317_T01	1
Ubiquitin-conjugating enzyme E2-17 kDa [b]	GRMZM5G814314_T04	4
Serine/Threonine protein kinases [b]	GRMZM2G043350_T01	1
OCS element-binding factor 1 [b]	GRMZM2G479885_T01	1
60S ribosomal protein	GRMZM2G158568_T01	56
Histone H2A	GRMZM2G305046_T01	5
NADH ubiquinone oxidoreductase B22-like subunit	GRMZM2G115621_T01	3
Glutathione S-transferase 2	GRMZM2G310031_T01	3
Exocyst complex subunit Sec15-like family protein	GRMZM5G885285_T01	2
Beta-4 tubulin (tub4)	GRMZM2G066191_T01	2
Thioredoxin-like protein 5	GRMZM2G382673_T01	2
Quinone reductase 2	GRMZM2G159643_T01	2
Coiled-coil domain-containing protein 25	GRMZM2G055898_T01	1
Transcription factor RF2b	GRMZM2G149040_T01	1
12-oxo-phytodienoic acid reductase 1	GRMZM2G106303_T01	1
Lipoxygenase (LOX11)	GRMZM2G009479_T01	1
Allene oxide cyclase 4	GRMZM2G077316_T01	1
Malate synthase1 (mas1)	GRMZM2G102183_T02	1
$Ca2^{+}/H^{+}$ exchanger	GRMZM2G011592_T01	1
Eukaryotic Lanthionine synthetase C-like protein	GRMZM2G703928_T02	1
Transferase, transferring glycosyl groups	GRMZM2G078890_T01	1
Beta3 tubulin (tub3)	GRMZM2G108766_T02	1
Putative ATP synthase	GRMZM2G351125_T01	1
Hypothetical protein	GRMZM2G134424_T03	1
ZM_BFb0154L01	GRMZM2G019328_T02	1
Phospholipid hydroperoxide glutathione	GRMZM2G135893_T01	1
Transposon protein Pong sub-class	GRMZM2G129508_T01	1
Hypothetical protein	GRMZM2G479340_T01	1
Phenazine biosynthesis protein	GRMZM2G050325_T01	1
Metal ion binding protein	GRMZM2G150450_T01	1
Nucleosome/chromatin assembly factor group A	GRMZM2G140051_T02	1
30S ribosomal protein S9	GRMZM2G024312_T01	1
Esterase	GRMZM2G170646_T01	1
Ascorbate peroxidase (POD1)	GRMZM2G137839_T01	1
Histone H2B.4	GRMZM2G112912_T01	1

Protein identification (cont.)	Ensembl.gramene.org accession No.	No. of positive clones
Histone H4	GRMZM2G016232_T01	1
Kinesin light chain	GRMZM2G074957_T01	1
Cysteine protease	GRMZM2G166281_T01	1
Leucine Rich Repeat family	GRMZM2G022298_T01	1
Catalytic/ hydrolase	GRMZM2G179432_T02	1
monodehydroascorbate reductase	GRMZM2G084881_T01	1
Glutathione S-transferase 29	GRMZM2G127789_T01	1

[a] Interactions highlighted in grey have been independently confirmed by BiFC analyses. For details see text.

[b] No interaction with RUM1 was identified by BiFC because protein expression could not be detected in Arabidopsis protoplasts by western blot.

Supplementary Table S4. Characteristics of the *rap1* gene family in maize.

Name	AC maizeGDB	Maize chromo-some	Genome location	Strand	Protein length (aa)	Sub-genome	Syntenic paralog in maize	Syntenic ortholog in rice	Syntenic ortholog in sorghum
rap1	GRMZM2G124317	9	123,407,990-123,411,206	-1	99	N/A	N/A	N/A	N/A
ral1	GRMZM2G172244	1	73,379,019-73,382,453	-1	106	1	N/A	LOC_Os03g30430	Sb01g032430
ral2	GRMZM2G017809	3	64,705,578 - 64,706,318	1	151	N/A	N/A	N/A	N/A
ral3	GRMZM2G165461	4	4,861,353-4,862,432	1	113	1	N/A	LOC_Os11g41150	Sb05g025170
ral4	GRMZM2G100662	8	51,237,461-51,237,706	-1	67	N/A	N/A	N/A	N/A
ral5	GRMZM2G127386	10	17,675,697-17,679,188	1	101	1	N/A	LOC_Os12g31780	Sb08g015560
ral6	GRMZM2G164340	2	15,872,003-15,872,927	-1	123	1	N/A	LOC_Os04g48870	Sb06g026180
